# Multifaceted Impact of CO_2_ Laser Therapy on Genitourinary Syndrome of Menopause, Vulvovaginal Atrophy and Sexual Function

**DOI:** 10.3390/healthcare12141385

**Published:** 2024-07-11

**Authors:** Svetlana Jankovic, Marija Rovcanin, Milena Zamurovic, Branka Jovanovic, Tatjana Raicevic, Ana Tomic

**Affiliations:** 1 Clinic for Gynecology and Obstetrics, Narodni Front, Kraljice Natalije 62, 11000 Belgrade, Serbia; 2 Faculty of Medicine, University of Belgrade, Dr Subotica Starijeg 8, 11000 Belgrade, Serbia; 3 Center for Radiology and Magnetic Resonance Imaging, University Clinical Center of Serbia, 11000 Belgrade, Serbia

**Keywords:** genitourinary syndrome, menopause, female sexual health, fractional CO_2_ laser

## Abstract

Genitourinary syndrome of menopause (GSM) encompasses a range of distressing symptoms in the vulvovaginal and/or bladder–urethral regions related to menopause changes, negatively influencing woman’s quality of life and sexual activity. Fractional micro-ablative CO_2_ laser therapy has shown the potential to reinstate the vaginal epithelium to a condition akin to the premenopausal state, thereby ameliorating the subjective symptoms associated with GSM. We conducted a prospective, pilot study in 73 sexually active postmenopausal women treated with CO_2_ laser for their GSM symptoms, while assessing Vaginal Health Index Score (VHIS) and sexual function through the Female Sexual Function Index (FSFI) Questionnaire. The laser treatment resulted in a decrease in VHIS and patient-reported vulvovaginal atrophy (VVA) symptoms, with a significantly lower prevalence of vaginal itching, dryness, and burning (*p* < 0.001), as well as dyspareunia (*p* = 0.002). The occurrence of urinary incontinence, urgency, and vaginal heaviness significantly reduced, with an improvement in the staging of cystocele, either to Stage 1 or complete resolution (*p* < 0.001). FSFI total and domain scores were significantly higher after the treatment, indicating better sexual function, with a post-treatment score median of 25 (*p* < 0.001). Therefore, using a three-cycle fractional CO_2_ laser was an effective choice for reducing urogenital discomfort related to GSM in postmenopausal women.

## 1. Introduction

Introduced a decade ago, genitourinary syndrome of menopause (GSM) encompasses a range of distressing symptoms in the vulvovaginal and/or bladder–urethral regions related to menopause changes [1]. Hence, GSM is not associated with any other medical conditions, as it is characterized by a menopause-induced decrease in sex steroids and entails alterations in the labia, clitoris, vestibule, vagina, urethra, and bladder [2]. GSM occurs in approximately 50% of menopausal women [3,4], characterized by its chronic and progressive nature [1]. The reluctance of both women and healthcare professionals to discuss issues related to vaginal health frequently results in underdiagnoses and undertreatment [1,4].

Women who are affected may experience symptoms that are associated with the vaginal tract, urinary tract, or both [4]. Genital symptoms are a manifestation of vulvovaginal atrophy (VVA), mostly consisting of vaginal dryness and pain during sexual intercourse, known as dyspareunia, arising due to a compromised response to sexual stimulation [4]. Prevalent urinary symptoms include frequency, urgency, dysuria and stress incontinence [2,4,5]. A number of surveys have provided comprehensive information regarding the adverse impacts of GSM on various aspects of menopausal women’s lives, including their quality of life, emotional well-being, and sexual functioning [6,7]. Not less than roughly 50% of menopausal women experience symptoms of GSM in regard to sexual desire, intimacy, well-being, and self-worth [7].

The global significance of vaginal laser therapy in managing genitourinary syndrome of menopause is slowly increasing [8]. The utilization of laser therapy has been found to enhance the vascularization of the vaginal mucosa, promoting the production of newly derived collagen and matrix basic substance within the connective tissue, supporting the thickening of the epithelium that lines the vagina through the formation of new papillae, as it facilitates the restoration of mucosal equilibrium, consequently alleviating the aforementioned symptoms associated with GSM [9]. Hence, fractional micro-ablative CO_2_ laser therapy has the potential to reinstate the vaginal epithelium to a condition akin to the premenopausal state, thereby ameliorating the subjective symptoms associated with GSM-associated VVA symptoms as well as lower urinary tract discomfort [10]. Moreover, it was observed that the CO_2_ laser could be effective for treating urinary GSM symptoms because of the improvement in atrophy of the urethral and bladder mucosa. Additionally, it could ultimately result in enhanced genital health and increased satisfaction with sexual life among postmenopausal women [11,12,13].

Laser and radiofrequency techniques are currently being extensively researched in order to have a full understanding of their overall efficacy and safety [5], with a scarcity of research that has explored the impact of this intervention on sexual function, as well as GSM-associated urinary discomfort. Therefore, the objective of this study was to evaluate the impact of a micro-ablative fractional CO_2_ laser on clinical symptoms of urinary and genital symptoms associated with GSM and concordant sexual function in postmenopausal women.

## 2. Materials and Methods

### 2.1. Study Design

This prospective, pilot study was conducted at our clinic between June and November of 2022 in order to evaluate GSM symptoms as well as concordant vaginal atrophy and sexual function after completing three cycles of laser treatment.

### 2.2. Participants

This study comprised a cohort of 73 postmenopausal women who were sexually active and experienced uncomfortable GSM symptoms resulting in sexual health complaints. The inclusion criteria included participants that had engaged in sexual activity within the past four weeks, with the absence of menstruation for at least one year, suffering at least one subjective GSM symptom (such as urinary incontinence, urgency, the presence of heaviness in vagina, vaginal itching, stinging, dyspareunia, dryness), and the presence of any severity of cystocele, diagnosed with VVA by a gynecologist. The exclusion criteria included the application/usage of any hormonal replacement therapy (HRT) (either systemic or local) within the past year, acute or recurrent urinary tract or genital infection, and suffering from hormonal imbalance or any serious disease, chronic condition, and or psychiatric disorders that could interfere with study compliance or which would prevent appropriate informed consent or study participation.

### 2.3. Data Collection and Intervention

The general questionnaire was administered to collect sociodemographic and anamnestic knowledge, including age, time since the last menstruation, past deliveries, and types of deliveries, prior to initiating the first laser application.

#### 2.3.1. Urinary Symptoms and Cystocele Staging

Data pertaining to the presence of urinary GSM symptoms (incontinence, urgency, the presence of heaviness in vagina) were collected through respondents’ answers to yes-or-no questions evaluated before starting the first laser application and 4 weeks after the third treatment. A cystocele, defined as a prolapse of the upper anterior vaginal wall involving the bladder was diagnosed and staged using the Pelvic Organ Prolapse Quantification System (POPQ), where stage 0 indicated no presence of prolapse, and stage 4 indicated complete vaginal eversion [14].

#### 2.3.2. VVA Symptoms and Vaginal Health Index Score (VHIS)

Prior to treatment and 4 weeks after the third laser treatment, women were evaluated by using the Vaginal Health Index score (VHIS) that assesses 5 characteristics of the vaginal wall: elasticity, fluid volume, pH, epithelial integrity, and moisture. The severity of each characteristic was evaluated based on the 5-point Likert scale, ranging from 1 to 5. The total score of VHI varies from 5 to 25 with its cut-off point of 15, as a score less than 15 indicates atrophic vaginitis [15]. The severity of VVA symptoms (vaginal burning, vaginal itching, vaginal dryness, and dyspareunia) was self-evaluated by study participants using a 10 cm visual analogue scale (VAS), where the value of 1 represented the absence of symptoms, while values 2–10 indicated the presence of symptoms with varying degrees of severity, as the value of 10 represents the most severe form. Incidence of all noted VVA symptoms (positive clinical findings) were defined by all responses greater than 1.

#### 2.3.3. Female Sexual Function Index (FSFI)

Prior to the initial laser treatment and four weeks after the third treatment, the assessment of sexual function was conducted using the Female Sexual Function Index Questionnaire [16]. The Female Sexual Function Index (FSFI) is an approved instrument assessing six domains of sexual function in women: desire, arousal, lubrication, orgasm, satisfaction, and pain as well as an overall score for sexual functioning (total FSFI). A cut-off score of 26.5 was used for the detection of Female Sexual Dysfunction [17].

#### 2.3.4. Fractional CO_2_ Laser Treatment

Postmenopausal women were treated intravaginally with the fractional micro-ablative CO_2_ laser system (SmartXide2 V2 LR, Monalisa Touch; DEKA, Florence, Italy), using the following settings: dot power 35 W, dwell time 1000 μs, dot spacing 1000 μm, and the smart stack parameter from 1 to 3. The vaginal probe was inserted and rotated along the vaginal canal, applying laser energy to the full length of the vagina. A complete treatment cycle included three laser applications, spaced 6 to 8 weeks apart which all participants completed. The patients bore the expense of the procedure’s treatment cost, which took place at the outpatient clinic without requiring any specific preparation or anesthesia.

### 2.4. Ethical Consideration

This study was implemented in accordance with the International Code of Medical Ethics of the World Medical Association (Declaration of Helsinki) and written informed consent was obtained from the participants after the nature and objectives of this study were fully explained to them. This study was approved by the institution’s Ethical Committee (date of approval: 16 January 2022).

### 2.5. Data Analysis

Data presented in the text and tables are reported as means ± standard deviation or frequencies (n) and accordant percentages (%). A McNemar test and Wilcoxon signed ranks test were used to define statistical significance of continuous indicators before/after the treatment variables. The Chi-squared test was employed to assess disparities across various patient groups based on their prior delivery approach. Spearman’s correlation analysis was employed to describe the relationships among two continuous variables. Statistical analysis was performed using IBM SPSS Statistics for Windows, version 23.0 (IBM Corp., Armonk, NY, USA). The significance level was set at *p* < 0.05. All 73 participants were included in this analysis.

## 3. Results

As presented in Table 1, our study involved 73 menopausal women, with mean deliveries of 55.6 ± 5.6 years old, with a menopause that averagely lasted for 6.6 ± 4.9 years. Our patients had a previous delivery in 63 (86.3%) cases, out of which 54 (74.0%) cases were vaginal deliveries, while 9 (12.3%) were deliveries by cesarean section.

### 3.1. Urinary GSM Symptms

We examined the extent to which urinary symptoms were completely alleviated following the laser therapy. As presented in Table 2, urinary incontinence, urgency, and heaviness in the vagina were significantly reduced (*p* < 0.001). After three cycles of laser treatment, the cystocele stage improved in a significant portion of our cohort (*p* < 0.001), to either stage 1 (37.0%) or was completely resolved. None of our patients exhibited a rectocele or a uterine prolapse.

### 3.2. Vulvovagnal Atrophy Symptoms and Sexual Function

According to the data presented in Table 3, we examined the extent to which VVA symptoms were completely alleviated following the laser therapy. Any absence of observed pain was regarded as a positive clinical observation. VHIS and patient-reported VVA symptoms show a significant decrease following the administration of CO_2_ laser treatment.

Upon examining the VHIS score, it was found that just 1 patient (1.4%) had a clinically relevant state of impaired vaginal health. Although the occurrence of VVA symptoms was diminished in a significant proportion of participants, a notable number of participants still reported experiencing a certain level of symptoms, particularly dyspareunia (74.0% of cases after the completed treatment) and vaginal dryness (69.9%).

As shown in Table 4, FSFI total and domain scores were significantly higher after the treatment, indicating better sexual function, with a total score median of 25.5, considered a clinically relevant level as compared to baseline median total score values (18.2).

When correlating VHIS and VVA clinical findings to FSFI domain and total scores, all significant correlations were mostly weak to moderate strength (Table 5). VHIS correlated positively with all domain scores, as well as the total FSFI score, suggesting that higher VHS is associated with better sexual function. However, VVA symptoms intensity correlation coefficients were negative, indicating an inverse relationship, where lower FSFI scores were associated with more pronounced symptoms.

Vaginal burning showed significant correlation to FSFI desire and lubrication domain scores, while dryness intensity significantly correlated to satisfaction domain scores. Intensity of dyspareunia was correlated to lubrication and pain domain scores, while being the only symptom to exhibit significant correlation to FSFI total score. Surprisingly, vulvovaginal itching was not significantly correlated to any of the domains or total score.

Ultimately, no adverse reactions were recorded by the patients.

According to the data presented in Table 6, our analysis of urinary and VVA symptoms revealed that there were no statistically significant variations in the prevalence of most analyzed symptoms between women who had a vaginal delivery and those who had a cesarean section. Statistically significant changes were seen only in the prevalence of urinary incontinence (*p* = 0.004) and urinary urgency (*p* = 0.014).

## 4. Discussion

Our study has shown that both urinary and VVA symptoms that were registered in menopausal women were alleviated following the laser therapy in a majority of the participants. After three cycles of laser treatment, cystocele stage improved in a significant portion of our cohort to either Stage 1 or was completely resolved. VHIS correlated positively with all domain scores, as well as the total FSFI score, suggesting that higher VHS is associated with better sexual function, while VVA symptoms indicated an inverse relationship.

Similar to our results, previous studies showed that the intravaginal fractional CO_2_ laser significantly improved urinary GSM symptoms such as nocturia and overall urinary frequency. Moreover, the mentioned authors reported a significant reduction in episodes of in women with urgency incontinence [18]. There are a small number of randomized control studies that tested the efficiency of CO_2_ laser for GSM symptoms compared to sham-laser treated controls. A double-blind, randomized, sham-controlled trial by Salvatore et al. [19] showed that laser-induced changes were in favor of the CO_2_-treated group, with a more pronounced effect for VVA symptoms like dryness, dyspareunia, and concomitant sexual dysfunction when compared to urinary symptoms such as dysuria. A study by Aguiar et al. [18] showed that only the intravaginal fractional CO_2_ laser significantly improved urinary GSM symptoms such as nocturia, frequency, and episodes of urgency when compared to other, topical treatments such as estrogens and lubricants. A Brazilian study by Politano et al. [20] showed that the use of fractional CO_2_ laser therapy to treat genitourinary syndrome resulted in better short-term effects than those of local estrogens or lubricant with respect to improving the VHIS score and overall vaginal health in postmenopausal women, with improvement in the desire and lubrication FSFI domains. Considering the regenerative impact of intravaginal fractional laser CO_2_ therapy, which extends to the lower urinary tract and leads to an improvement of menopausal urogenital symptoms, it has been proposed as a potentially effective option for addressing GSM involving urinary symptoms [18].

Nevertheless, the literature presents varying outcomes about the efficacy of laser treatment in alleviating urinary symptoms. A study by Page et al. [21] reported that the laser treatment response was comparable to that of sham applications, as there were no obvious differences in observed outcomes, with no improvement when it came to both sexual and urinary functions. Similar results were reported when CO_2_ was tested for GSM symptoms in breast cancer survivors [22]. Regrettably, a considerable amount of the existing literature does not provide sufficient evidence to support the superiority of laser treatment in alleviating GSM symptoms, as three-cycle laser treatment demonstrated comparable efficacy to other treatment options. Both fractionated CO_2_ vaginal laser and vaginal estrogen treatment showed comparable efficacy in improving symptoms of genitourinary syndrome of menopause, as well as urine and sexual function, 6 months post-treatment [23]. Similarly, recognized GSM treatment modalities, such as laser, radiofrequency, and vaginal estrogen yielded similar and considerable enhancements in GSM symptoms for women with breast cancer, a highly vulnerable group who were undergoing adjuvant therapy for their disease. While laser therapy and topical estrogens have different mechanisms of action, data suggest that laser therapy is an equally effective approach for addressing the subjective discomfort caused by urogenital atrophy [24], with an improvement in sexual function and overall quality of life [25].

Nonetheless, while contemplating the assessment of treatments, it may be paramount to prioritize the assessment of lasers over hormone treatments due to health concerns associated with the latter [26]. When compared to vaginal estrogens or fractional radiofrequency, it was estimated that CO_2_ laser was an adequate treatment alternative for noted treatment options, as hormonal treatment carries certain risk, while radiofrequency is rather often accompanied by pain [27]. Regarding hormone treatment, the lack of understanding of long-term follow-ups and limited data on the specific type, dosage, and method of administering hormones such as estrogens is particularly emphasized, as it has been observed that oral estrogens can exacerbate type-independent urinary incontinence in menopausal women [18]. What may set CO_2_ laser treatment apart from other noted treatment modalities is patient satisfaction and adherence to the treatment cycles. Regarding the patient’s overall impression, a study by Paraiso et al. [23] reported that 85.8% of participants rated their improvement as “better or much better” and 78.5% reported being either “satisfied or very satisfied” compared to 70% and 73.3% in the estrogen group. This is likely attributed to the enhanced laser treatment adherence, as the desired outcomes can be achieved in just three cycles with authors reporting that all patients who underwent the laser therapy successfully completed the whole treatment [28]. Furthermore, all the reported heterogenic results for the laser efficiency, we would like to emphasize that none of the above cited trials reported serious adverse effects, as it was the case in our study.

Prior research has observed that symptoms of VVA exhibit a roughly linear correlation with overall sexual functioning [29]. Accordingly, our analysis demonstrated a noteworthy enhancement in all FSFI domain scores following the treatment, consistent with the results previously reported by other authors [12,28,30]. Authors utilizing laser treatment showed significant improvement of total FSFI score and individual domains of desire and lubrication [20,31]. Moreover, literature-reported results showed that the improvement in the “Lubrication” domain of FSFI was only substantial when a laser with additional moisturizers was used [32]. However, some authors reported significant worsening of the pain FSFI domain [31]. It is imperative to carefully observe pain levels after laser procedures, as studies suggest that pain is the most influential element in predicting sexual functionality, compared to other domains whose effects contribute to overall sexual activity to a lesser extent [29]. According to our data, the pain domain exhibited greater scores in the presence of more pronounced dyspareunia, hence the highlighted post-treatment improvement of dyspareunia which may be imperative for optimal results for sexual function in menopause. Additionally, dyspareunia was the only subjective symptom that showed a significant correlation with the overall FSFI score, underscoring the significance of addressing and ameliorating this symptom of VVA.

Despite the compelling nature of our findings, some limitations should be considered. Compared to certain cited studies, our study lacks randomization and did not include a control group. However, it is important to mention that our study encompassed a wider range of investigated urinary GSM symptoms, such as urgency, frequency, heaviness in vagina, as well as the efficiency of laser treatment on the urinary organ prolapse-cystocele. Nevertheless, available research is still limited, as more randomized controlled studies in particular are lacking. Most of available studies show a trend toward safe and effective treatment, but only in the short-term [8]. Therefore, a significant drawback of this study is that we did not examine the long-term results beyond the four-week period after treatment. This prevents us from comprehending the long-term efficiency and any delayed effects associated with CO_2_ laser therapy. Although laser therapy for the treatment of the symptoms of GSM appears promising, there is currently a lack of high-level and long-term evidence regarding its safety and efficacy. Moreover, there is a lack of professional guidelines regarding this modality of treatment, specifically for GSM [10]. Opportunities exist for future research in this area, specifically to determine safety and long-term outcomes of therapy.

## 5. Conclusions

Our study found that using a three-cycle fractional CO_2_ laser treatment is an effective choice for reducing genital discomfort related to vulvovaginal atrophy in postmenopausal women. Furthermore, it had a notable impact in reducing urinary symptoms. Subsequently, the improvements led to an increase in scores related to overall sexual functioning, as well as certain domains. Although these findings show promising possibilities for future therapeutic use, further research is required to evaluate its long-term efficacy, potential adverse effects, and safety considerations. Furthermore, a potential source of bias in our study is the relatively small number of participants, which could restrict the applicability of our findings and impact the strength of the statistical analysis.

## Figures and Tables

**Table 1:** Patient characteristics.

Age (years)	55.6 ± 5.6
Menopause Duration (years)	6.6 ± 4.9
Previous Deliveries	63 (86.3%)
Previous Vaginal Delivery	54 (74.0%)
Previous Cesarean Section	9 (12.3%)

Values are presented as mean ± SD or frequencies and percentages (%).

**Table 2:** Urinary GSM symptoms at baseline level and after treatment with CO_2_ laser.

Positive Clinical Finding	Baseline	After Completed Treatment	*p* Value *
Urinary Incontinence	48 (65.8%)	16 (21.9%)	<0.001
Urinary Urgency	45 (61.6%)	21 (28.8%)	<0.001
Vaginal Heaviness	34 (46.6%)	12 (16.4%)	<0.001
Cystocele (POPQ Staging)			
Stage 1	10 (13.7%)	27 (37.0%)	<0.001
Stage 2	24 (32.9%)	3 (4.1%)	
Stage 3	7 (9.6%)	0 (0%)	

POPQ—Pelvic Organ Prolapse Quantification System; data are expressed as n (%); * obtained with a McNemar test or Wilcoxon signed rank test when appropriate; significant at <0.05.

**Table 3:** Vulvovaginal atrophy symptoms presence at baseline level and after treatment.

Positive Clinical Finding	Baseline	After Completed Treatment	*p*-Value **
VVA (VHIS < 15)	54 (74.0%)	1 (1.4%)	<0.001
Vaginal Itching *	51 (69.9%)	30 (41.1%)	<0.001
Vaginal Burning *	55 (75.3%)	33 (45.2%)	<0.001
Vaginal Dryness *	71 (97.3%)	51 (69.9%)	<0.001
Dyspareunia *	64 (87.7%)	54 (74.0%)	0.002

VVA—vulvovaginal atrophy; VHIS—Vaginal Health Index Score; data are expressed as n (%); * VA Scale higher than 1 was equivalent to the presence of symptoms and positive clinical finding; ** obtained with McNemar test, significant at <0.05.

**Table 4:** FSFI scores before and after treatment with CO_2_ laser.

FSFI Scores	Before Treatment	After Treatment	*p*-Value *
Total	18.2 ± 6.5	25.5 ± 4.1	<0.001
Desire	3.0 ± 1.1	4.1 ± 0.8	<0.001
Arousal	3.0 ± 1.2	4.2 ± 0.8	<0.001
Lubrication	2.8 ± 1.3	4.2 ± 0.8	<0.001
Orgasm	3.2 ± 1.3	4.4 ± 0.9	<0.001
Satisfaction	3.3 ± 1.3	4.2 ± 1.0	<0.001
Pain	2.9 ± 1.5	4.3 ± 0.9	<0.001

FSFI—Female Sexual Function Index; * Values are presented as mean ± SD; * Wilcoxon signed rank test, significant at *p* < 0.05.

**Table 5:** Correlation coefficients.

	Desire	Arousal	Lubrication	Orgasm	Satisfaction	Pain	Total
VHIS	0.348 **	0.349 **	0.490 **	0.332 **	0.310 **	0.513 **	0.496 **
Vaginal Itching *	−0.145	0.081	−0.033	0.059	−0.028	−0.041	−0.044
Vaginal Burning *	−0.310 **	−0.008	−0.275 *	0.042	−0.010	−0.207	−0.209
Vaginal Dryness *	−0.043	−0.084	−0.206	0.028	−0.347 **	−0.220	−0.151
Dyspareunia *	−0.070	−0.196	−0.352 **	−0.059	−0.198	−0.427 **	−0.350 **

VHIS—Vaginal Health Index Score; Values are presented as spearman correlation coefficients; significant correlation coefficients are in bold; * *p* < 0.05; ** *p* < 0.001.

**Table 6:** Urinary and vulvovaginal atrophy symptoms presence depending on the previous delivery type.

Positive Clinical Finding	Vaginal Deliveries (*n* = 54)	Cesarean Section (*n* = 9)	*p*-Value **
Urinary Incontinence	43 (79.6%)	3 (33.3%)	0.004 *
Urinary Urgency	31 (57.4%)	9 (100.0%)	0.014 *
Vaginal Heaviness	30 (55.6%)	3 (33.3%)	0.217
Cystocele			
Stage 1	8 (14.8%)	1 (11.1%)	0.292
Stage 2	22 (40.7%)	2 (22.2%)	
Stage 3	6 (11.1%)	1 (11.1%)	
VVA (VHIS < 15)	37 (68.5%)	2 (22.2%)	0.575
Vaginal Itching *	40 (74.1%)	6 (66.7%)	0.643
Vaginal Burning *	41 (75.9%)	8 (88.9%)	0.386
Vaginal Dryness *	52 (96.3%)	9 (100.0%)	0.557
Dyspareunia *	45 (83.3%)	9 (100.0%)	0.186

VVA—vulvovaginal atrophy; VHIS—Vaginal Health Index Score; data are expressed as n (%); * VA Scale higher than 1 was equivalent to the presence of symptoms and positive clinical finding; ** obtained with Chi-squared test, significant at <0.05.

## Data Availability

The data presented in this study are available on request from the corresponding author.
